# Infection after primary hip arthroplasty: A comparison of 3 Norwegian health registers

**DOI:** 10.3109/17453674.2011.636671

**Published:** 2011-11-25

**Authors:** Håvard Dale, Inge Skråmm, Hege L Løwer, Hanne M Eriksen, Birgitte Espehaug, Ove Furnes, Finn Egil Skjeldestad, Leif I Havelin, Lars B Engesæter

**Affiliations:** ^1^The Norwegian Arthroplasty Register, Department of Orthopaedic Surgery, Haukeland University Hospital, Bergen; ^2^Department of Orthopaedic Surgery, Akershus University Hospital, Lørenskog; ^3^Norwegian Institute of Public Health, Oslo; ^4^Department of Surgical Sciences, Faculty of Medicine and Dentistry, University of Bergen, Bergen, Norway

## Abstract

**Background and purpose:**

The aim of the present study was to assess incidence of and risk factors for infection after hip arthroplasty in data from 3 national health registries. We investigated differences in risk patterns between surgical site infection (SSI) and revision due to infection after primary total hip arthroplasty (THA) and hemiarthroplasty (HA).

**Materials and methods:**

This observational study was based on prospective data from 2005–2009 on primary THAs and HAs from the Norwegian Arthroplasty Register (NAR), the Norwegian Hip Fracture Register (NHFR), and the Norwegian Surveillance System for Healthcare–Associated Infections (NOIS). The Norwegian Patient Register (NPR) was used for evaluation of case reporting. Cox regression analyses were performed with revision due to infection as endpoint for data from the NAR and the NHFR, and with SSI as the endpoint for data from the NOIS.

**Results:**

The 1–year incidence of SSI in the NOIS was 3.0% after THA (167/5,540) and 7.3% after HA (103/1,416). The 1–year incidence of revision due to infection was 0.7% for THAs in the NAR (182/24,512) and 1.5% for HAs in the NHFR (128/8,262). Risk factors for SSI after THA were advanced age, ASA class higher than 2, and short duration of surgery. For THA, the risk factors for revision due to infection were male sex, advanced age, ASA class higher than 1, emergency surgery, uncemented fixation, and a National Nosocomial Infection Surveillance (NNIS) risk index of 2 or more. For HAs inserted after fracture, age less than 60 and short duration of surgery were risk factors of revision due to infection.

**Interpretation:**

The incidences of SSI and revision due to infection after primary hip replacements in Norway are similar to those in other countries. There may be differences in risk pattern between SSI and revision due to infection after arthroplasty. The risk patterns for revision due to infection appear to be different for HA and THA.

Increasing incidence of revision due to infection after primary total hip arthroplasty (THA) has been observed in different countries during the last decade (Kurtz et al. 2008, Dale et al. 2009, Pedersen et al. 2010). There have been several studies on incidence of and risk factors for infection based on data from surveillance systems (Ridgeway et al. 2005, Mannien et al. 2008), arthroplasty (quality) registers (Berbari et al. 1998, Dale et al. 2009, Pedersen et al. 2010), and administrative databases (Mahomed et al. 2003, Kurtz et al. 2008, Ong et al. 2009). There have been reviews on incidence of and risk factors for infection after hip arthroplasty, based on publications from databases with different definitions of infection (Urquhart et al. 2009, Jämsen et al. 2010a). Superficial surgical site infections (SSIs) may have risk factors that are different from those of full surgical revisions due to infection. Furthermore, THA and hip hemiarthroplasty (HA) may have different patterns of risk of infection (Ridgeway et al. 2005, Cordero–Ampuero and de Dios 2010).

In the present study, we used data from 3 national health registries in Norway to assess incidence and some risk factors for infection after primary hip arthroplasty. Differences in risk patterns between SSI and revision due to infection were investigated for HA and THA.

## Material and methods

In Norway, 3 national health registries representing 2 different surveillance systems record information on primary hip replacement surgery and postoperative infections: the Norwegian Arthroplasty Register (NAR) and the Norwegian Hip Fracture Register (NHFR). These are quality registers, while the Norwegian Surveillance System for Healthcare–Associated Infections (NOIS (Norwegian acronym)) is an infection surveillance system. We compared these registries for infectious endpoints after primary THA or HA over the years 2005–2009. In addition, data from a fourth health registry, the Norwegian Patient Register (NPR), were used to assess the reporting of primary procedures to the 3 registries.

### The Norwegian Surveillance System for Healthcare–Associated Infections (NOIS)

The NOIS is based on a modified version of Hospitals in Europe Link for Infection Control through Surveillance (HELICS 2004). The aims are to survey, describe, and evaluate the incidence of surgical site infection (SSI) after certain procedures. Furthermore, the intention is to assess effects of prophylactic interventions and discover variations in SSI. Since 2005, it has been mandatory for all Norwegian hospitals to report arthroplasty and 4 other procedures (Caesarean section, coronary bypass, appendectomy, and cholecystectomy) over a 3–month period every year (September to November). The data are collected either electronically from the patients' medical records or manually (by infection–control nurses) into a standardized case report form. The information collected includes hospital affiliation, patient characteristics, date of admission, surgery, discharge, first infection and last follow–up, type of arthroplasty, type of infection, the source of diagnosis (patient or physician), and reoperations. For this study, only infections verified by a medical doctor were included. Verification of SSI was from a form signed by a general physician or from the hospital medical records if the patient had SSI diagnosed at a hospital. The endpoint in the NOIS was SSI, defined according to the CDC guidelines. The CDC–defined organ/space SSI category was combined with the deep incisional SSI category. Reoperations reported to the NOIS comprised all types of surgical procedures due to infection. If no infection was recorded, the patient was censored at death or last date of surveillance. Endpoint evaluation was done at discharge, by questionnaire to the patient, and by evaluation of the medical records at 30 and 365 days postoperatively. 30 days were defined as the minimum follow–up time for inclusion. The procedures included were primary THAs and HAs with the NOMESCO codes NFB 02, –12, –20, –30, and –40. In the NOIS, 6,956 hip arthroplasties, including 5,540 THAs and 1,416 HAs, were eligible for analysis (Figure 1). In contrast to the NHFR, the NOIS also includes HAs inserted for causes other than femoral neck fracture. With this exception, THAs in the NOIS should also be reported to the NAR whereas HAs should be reported to the NHFR.

**Figure:**
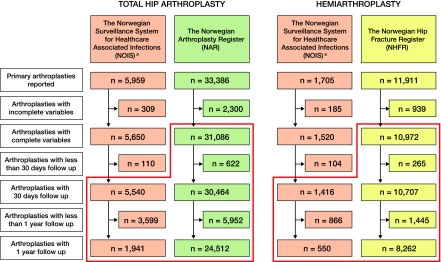
Number of primary THAs and HAs (red squares) included in the NOIS, the NAR, and the NHFR, including number of arthroplasties with missing data on the confounders and incomplete 30–day and 1–year follow–up. **^a^** The NOIS registers arthroplasties 3 months every year. Not all hospitals that reported to the NAR and the NHFR reported to the NOIS. (NOIS is a Norwegian acronym).

### The Norwegian Arthroplasty Register

Since its inception in 1987, the NAR has registered data on primary THAs and THA revisions. This includes the patient's identity and characteristics, the indication for THA, the surgical procedure, the implant, and revisions. The unique identification number of each Norwegian citizen can be used to link the primary THA to a later revision (Havelin et al. 2000).

Revision due to deep infection of the implant was the infection endpoint in the NAR in the present study, and was defined as removal or exchange of the whole or part of the prosthesis with deep infection reported as the cause of revision. Isolated soft tissue revisions were not reported. The case report form is filled in by the surgeon immediately after surgery. Detailed information on the arthroplasty was transformed into the following NOMESCO groups: cemented THAs (NFB 40), uncemented THAs (NFB 20), and hybrid THAs (NFB 30). The NAR does not register HAs. All THAs were followed until their first revision due to deep infection or revision for other causes, until the date of death or emigration of the patient, or until December 31, 2009. In the NAR, 31,086 THAs were eligible for analysis (Figure).

### The Norwegian Hip Fracture Register

The NHFR has the same administrative foundation and purpose as the NAR. Since January 1, 2005, all hip fractures treated surgically and later revisions have been reported on a similar case report form to that for registration in the NAR (Gjertsen et al. 2008). Procedures included were HAs performed as a primary operation for a femoral neck fracture and HAs inserted secondary to failure of the primary osteosynthesis of a femoral neck fracture. THAs as primary emergency treatment or secondary planned treatment of femoral neck fractures were registered in the NAR. As for the NAR, the endpoint was revision of the implant due to infection. The groups cemented HA (NFB 12) and uncemented HA (NFB 02) were defined based on detailed information about the implant type and fixation reported to the NHFR. HAs inserted for causes other than hip fracture or complications after hip fracture (i.e. osteoarthritis or malignancies) were not registered in the NHFR. All HAs were followed until their first revision due to deep infection or revision for other causes, until the date of death or emigration of the patient, or until December 31, 2009. In the NHFR, 10,972 HAs were eligible for analysis (Figure).

### The Norwegian Patient Register

The NPR is a national administrative health register. It is compulsory by law to report medical treatment to the NPR, and it is the basis of funding in Norwegian hospitals. Primary THAs and HAs with the NOMESCO codes NFB 02, –12, –20, –30, and –40 were included for the assessment of case reporting, regardless of diagnosis.

### Statistics

Descriptive statistics were used for demographics and surgery–related data. Data from NOIS and the merged NAR and NHFR data were analyzed separately. The 1–year incidences of SSI, reoperation, and revision due to infection were estimated by dividing the number of events reported during the first postoperative year by the number of primary arthroplasties. Cox regression analyses were performed to establish risk factors for revision due to deep infection or SSI, and also 1–year probabilities (risks) of these events (1 minus 1–year survival (%)). Adjusted hazard rate ratios, hereafter called risk ratios (RRs), were estimated for each risk factor with 95% confidence intervals (CIs). The risk factors evaluated were age, sex, American Society of Anesthesiologists classification (ASA class), duration of surgery, type of surgery (emergency or planned), and method of fixation of the prosthesis (Table 1). Adjusted risk of SSI after HA relative to THA was assessed in the NOIS, whereas adjusted risk of revision due to infection after HA relative to THA was analyzed in the merged NAR–NHFR data. In addition, we calculated and assessed the National Nosocomial Infection Surveillance (NNIS) risk index, which comprise duration of surgery (> 75th percentile for the procedure), co–morbidity of the patient (ASA class > 2), and contamination of the wound peroperatively (Mangram et al. 1999). In the NAR and NHFR, we assumed that there was no contamination. The 75 percentile duration of surgery as reported was used (in the NOIS, HA 94 min and THA 108 min; in the NHFR, HA 90 min; and in the NAR, THA 110 min), and not the 120 min estimated for HA and THA in the HELICS guidelines. Follow–up for the NAR and the NHFR analyses was 0–5 years and for the NOIS it was 0–1 year. However, to ensure similar follow–up for all 3 registries, additional analyses were performed with follow–up restricted to 1 year for all available cases. Stratified analyses were performed on the NOIS data with deep SSI as separate endpoint. Any p–values of less than 0.05 were considered statistically significant. SPSS software version 18.0 and PASS 2008 software were used for statistical analysis.

**Table 1.** Distribution of the assessed risk factors in the registers: THAs and HAs included from the Norwegian Surveillance System for Healthcare Associated Infections (NOIS), THAs included from in the Norwegian Arthroplasty Register (NAR) and HAs included from in the Norwegian Hip Fracture Register (NHFR).

	Total hip arthroplasty		Hemiarthroplasty	
Register	NOIS	NAR	NOIS	NHFR
Number of procedures	5,540	31,086	1,416	10,972
Risk factor %				
Agegroup (years)				
<60	19	20	2	1
60–69	31	30	4	5
70–79	34	34	22	24
80–89	15	15	56	54
≥90	1	1	16	15
Sex				
female	66	67	74	74
male	34	33	26	26
ASA score				
1	18	29	2	5
2	65	52	42	35
≥3	17	19	56	60
Duration of surgery (Min)				
<60	6	7	19	22
60–89	41	40	50	47
90–119	33	35	22	24
≥120	19	19	8	8
Type of surgery				
emergency	3	2	74	86
planned	97	98	26	14
Method of fixation				
cemented	64	65	81	81
uncemented	16	16	19	19
hybrid	20	18		
NNIS index				
0	63	64	32	31
1	32	31	54	56
≥2	5	5	14	13

## Results

### Case reporting and distribution of risk factors

32 hospitals reported THAs to the NPR and the NAR during the study period. 30 hospitals reported THAs to the NOIS; there was an increase in hospitals reporting THA, from 8 in the first year.

29 hospitals reported HAs to the NPR, whereas 27 reported HAs to the NHFR and 26 reported HAs to the NOIS over the study period. The number of hospitals reporting HAs increased from 5 to 26 in the NOIS and from 26 to 27 in the NHFR. 33,466 primary THAs and 12,069 primary HAs were reported to the NPR during the study period. The comparable number of procedures reported to each of the other registries is presented in Figure 1. The distribution of risk factors was similar for THAs in the NOIS and the NAR, and for HAs in the NOIS and the NHFR (Table 1). The exception was ASA classification (Table 1).

### Incidence and risk of infection

The 1–year incidence of SSI was 3.0% after primary THA (Table 2). 6/94 of the superficial SSIs and 52/73 of the deep SSIs after THA in the NOIS were reported to have been reoperated due to the infection, whereas in the NAR the 1–year incidence of revision due to infection was 0.7% (Table 2).

**Table 2.** 1-year incidence of SSI and reoperation after primary arthroplasties as reported to the Norwegian Surveillance System for Healthcare Associated Infections (NOIS) and 1-year incidence of revisions due to infection after THAs as reported to the Norwegian Arthroplasty Register (NAR) and after HAs as reported to the Norwegian Hip Fracture Register (NHFR)

Register**^a^**	Endpoint	Total hip arthroplasty 1–year incidence	Hemiarthroplasty 1–year incidence
NOIS			
Sugical Site Infection		3.0% (167/5,540)	7.3% (103/1,416)
Superficial SSI		1.7% (94/5,540)	2.2% (31/1,416)
Deep SSI		1.3% (73/5,540)	5.1% (72/1,415)
SSI reoperated		1.0% (58/5,540)	3.6% (51/1.416)
NAR / NHFR			
Revision due to infection		0.7% (182/24,512)	1.5% (128/8,262)

**^a^** The NOIS and the NAR/NHFR represents different selections of cases

In primary HAs, the 1–year incidence of SSI was 7.3% (Table 2). 50/51 of the reoperations due to infection after HA in the NOIS were due to deep SSIs, and 50/72 of the deep SSIs were reported to have been reoperated. 1.5% of the HAs were reported to the NHFR to have been revised due to infection (Table 2).

In the NOIS, the adjusted risk of SSI after HA compared to THA was 1.2 (CI: 0.8–1.9). The adjusted risk of revision due to infection was 1.8 times higher for HAs (CI: 1.2–2.7) than for THA in the merged NAR/NHFR data.

### Time to SSI and revision due to infection

For THAs, the median postoperative time to diagnosis of SSI was 16 (2–214) days. Median time to revision due to infection was 29 (4–343) days, when restricting follow–up to 1 year, and 47 (4–1,782) days with 0–5 years of follow–up. For HAs, the median postoperative time to SSI was 15 (2–79) days. Median time to revision due to infection was 20 (4–304) days with 1–year follow up and 20 (4–701) days with 0–5 years of follow–up. 72% of the SSIs in the NOIS were identified in the post discharge surveillance, but only 9 cases of SSI were identified between 30 and 365 days postoperatively.

### Risk factors for infection after THA

The following factors were associated with increased risk of revision due to infection: 70–89 years of age, male sex, and ASA class higher than 1 (Table 3). Emergency surgery as opposed to planned surgery and a National Nosocomial Infection Surveillance Systems (NNIS) risk index of > 1 were also associated with a higher risk of revision due to infection after THA. Uncemented fixation of the prosthesis had a 50% higher risk of revision due to infection compared to cemented THAs.

**Table 3.** Risk factors for infection after THA: Adjusted risk of surgical site infection (SSI) after primary THAs in the Norwegian Surveillance System for Healthcare Associated Infections (NOIS), and adjusted risk of revision due to infection in the Norwegian Arthroplasty Register (NAR) for different risk factors. Each risk factor was adjusted for the other risk factors in the table except NNIS index

	Total hip arthroplasty											
Register: Number infected / included:	NOIS 167 / 5,540 (3.0%)						NAR 236 / 31,086 (0.8%)					
Risk factor	A	B	C	D	E	F	G	H	I	J	K	L
Age group (years)												
< 60	1,067	23	1			2.4	6,114	34	1			0.4
60–69	1,740	46	1.2	0.7–2.1	0.5	2.7	9,320	61	1.3	0.8–1.9	0.3	0.4
70–79	1,882	59	1.4	0.8–2.4	0.2	3.3	10,703	95	1.7	1.1–2.6	0.02	0.6
80–89	816	36	1.9	1.1–3.5	0.03	4.2	4,766	45	1.8	1.1–3.0	0.02	0.8
≥90	35	3	3.8	1.1–13	0.04	7.4	183	1	1.0	0.1–7.4	1.0	0.5
Sex												
female	3,676	103	1			2.7	20,922	113	1			0.4
male	1,864	64	1.3	0.9–1.8	0.1	3.6	10,164	123	2.4	1.8–3.1	<0.001	1.0
ASA score												
1	1,010	18	1			2.1	8,964	46	1			0.4
2	3,608	109	1.5	0.9–2.5	0.1	3.1	16,148	125	1.5	1.1–2.2	0.02	0.6
≥3	922	40	1.9	1.0–3.4	0.04	4.1	5,974	65	2.0	1.3–2.9	0.001	0.7
Duration of surgery (min)												
<60	357	20	2.4	1.4–4.0	0.001	6.8	2,045	15	1.0	0.6–1.8	0.9	0.6
60–89	2,294	56	1			2.4	12,427	84	1			0.5
90–119	1,822	59	1.3	0.9–1.9	0.1	3.2	10,745	84	1.1	0.8–1.5	0.5	0.5
≥120	1,067	32	1.2	0.8–1.9	0.4	3.2	5,869	53	1.3	0.9–1.8	0.2	0.5
Type of surgery												
emergency	183	10	1.8	0.9–3.4	0.08	6.6	609	9	2.2	1.1–4.3	0.02	1.3
planned	5,357	157	1			2.9	30,477	227	1			0.5
Method of fixation												
cemented	3,547	111	1			2.9	20,308	159	1			0.5
unemented	902	25	1.0	0.7–1.7	0.8	3.5	5,110	43	1.5	1.0–2.2	0.03	0.8
hybrid	1,091	31	1.1	0.7–1.7	0.7	3.4	5,668	34	1.1	0.7–1.6	0.7	0.6
NNIS index **^a^**												
0	3,480	94	1			2.8	19,729	129	1			0.5
1	1,784	64	1.3	0.9–1.7	0.2	3.8	9,760	87	1.3	1.0–1.7	0.08	0.6
≥2	267	9	1.0	0.5–2.0	1.0	3.5	1,597	20	1.7	1.1–4.4	0.02	0.6

A Number of primary arthroplasties included B Number of SSIs C Adjusted risk of SSI D 95% CI E P-value F Adjusted 1–year SSI percent G Number of primary THAs included H Number of revisions due to infection I Adjusted risk of revision due to infection J 95% CI K P-value L Adjusted 1–year revision percent

**^a^** Adjusted for sex, age, type of surgery and method of fixation

Risk factors for SSI after THA were short duration of surgery (< 60 min), which was also the finding when cemented, uncemented, and hybrid fixations were analyzed separately. Patients older than 80 years of age also had higher risk of SSI than those who were less than 60 years of age. The risk patterns for SSI and revision due to infection were different regarding effects of gender, duration of surgery < 60 min, and method of fixation. Separate analyses of all cases with 1–year follow–up in the NOIS and restricted follow–up of 1 year for the NAR as in the NOIS did not change the findings concerning risk factors for SSI or revision due to infection. Restriction to deep incisional SSI in the NOIS had only minor effects on the risk estimates.

### Risk factors for infection after HA

In the NHFR, age less than 60 years and duration of surgery of less than 60 min were associated with increased risk of revision due to infection (Table 4). No risk factors were identified for SSI after HA. HA had a different risk profile from that of THA, for both SSI and revision due to infection (Tables 3 and 4).

**Table 4.** Risk factors for infection after HA: Adjusted risk of surgical site infection (SSI) after primary HAs in the Norwegian Surveillance System for Healthcare Associated Infections (NOIS), and adjusted risk of revision due to infection in the Norwegian Hip Fracture Register (NHFR) for different risk factors. Each risk factor was adjusted for the other risk factors in the table except NNIS index

	Hemiarthroplasty											
Register: Number infected / included:	NOIS 103 / 1,416 (7.3%)						NHFR 167 / 10,972 (1.5%)					
Risk factor	A	B	C	D	E	F	G	H	I	J	K	L
Age group (years)												
<60	22	1	0.8	0.1–5.8	0.8	4.0	145	7	3.6	1.6–7.8	0.001	5.1
60–69	51	4	1.2	0.4–3.4	0.7	6.7	566	8	1.0	0.5–2.1	1.0	1.4
70–79	318	19	1.0	0.6–1.6	0.9	6.0	2,634	41	1.1	0.8–1.7	0.5	1.6
80–89	796	54	1			6.2	5,946	80	1			1.4
≥90	229	25	1.6	1.0–2.6	0.06	8.7	1,681	31	1.4	1.0–2.2	0.08	2.1
Sex												
Female	1,053	82	1			7.0	8,085	115	1			1.5
Male	363	21	0.8	0.5–1.3	0.3	4.8	2,887	52	1.3	1.0–1.9	0.08	2.0
ASA score												
1	25	0					523	7	1			1.3
2	592	43	1			7.3	3,854	58	1.2	0.5–2.6	0.6	1.5
≥3	799	60	1.1	0.7–1.6	0.8	8.2	6,595	102	1.3	0.6–2.8	0.5	1.6
Duration of surgery (min)												
<60	271	26	1.9	1.0–3.9	0.06	7.8	2,371	47	1.4	0.9–2.0	0.1	2.2
60–89	709	53	1.7	0.9–3.2	0.08	7.2	5,152	77	1			1.6
90–119	317	13	1			3.7	2,598	30	0.8	0.5–1.2	0.2	1.2
≥120	119	11	2.2	1.0–4.9	0.06	8.0	851	13	0.9	0.5–1.7	0.7	1.4
Type of surgery												
Emergency	1,041	81	1.3	0.8–2.0	0.8	6.9	9,459	137	0.8	0.5–1.1	0.2	1.5
Planned	375	22	1			5.4	1,513	30	1			2.0
Method of fixation												
Cemented	1,141	74	1			6.0	8,849	127	1			1.5
Unemented	275	29	1.4	0.9–2.3	0.1	8.5	2,123	40	1.2	0.8–1.7	0.4	1.8
NNIS index **^a^**												
0	452	32	1			7.2	3,436	54	1			1.6
1	759	56	1.1	0.7–1.7	0.7	8.2	6.113	92	1.0	0.7–1.4	1.0	1.6
≥2	190	15	1.2	0.6–2.2	0.6	7.9	1.423	21	0.9	0.6–1.6	0.8	1.5

A–L: See table 3.

**^a^** Adjusted for sex, age, type of surgery and method of fixation

## Discussion

The 3.0% incidence of SSI after primary THA is similar to incidences of SSI reported from other European countries with similar surveillance systems to those of Norway (range 0.9–4.6%) (Ridgeway et al. 2005, HELICS 2006, The Health Protection Agency 2007, Mannien et al. 2008). The 1–year incidence of revision due to infection (0.7% for THA) in the NAR is similar to results from other Scandinavian arthroplasty registries (Havelin et al. 2009). Comparisons of incidence of infection after arthroplasty across countries are complicated due to differences in definitions, in completeness of case reporting, and in post–discharge surveillance (Wilson et al. 2007).

The 1–year incidence of SSI of 7.3% after primary HA appears to be high compared to the results reported from the English mandatory surveillance (3.6–5.0%), which has also reported that HA patients had 2.5 times greater risk of developing SSI than THA patients (Ridgeway et al. 2005, The Health Protection Agency 2007). Similar differences between SSI after HA and SSI after THA were also reported by Wilson from the HELICS collaboration (2007). One explanation for the higher infection rates after HA may be differences in patient population, including how frail individuals are from a medical standpoint (Gjertsen et al. 2008, Hahnel et al. 2009). HA patients were generally older, with more co–morbidity than the THA patients, and the majority of HA patients had had surgery due to a trauma (hip fracture). Ridgeway found, as in the present study, that there was no difference in the risk of SSI between HA and THA patients after adjusting for ASA score, age, duration of surgery, and procedures performed after trauma. In contrast, we found an increased risk of revision due to infection after HA as compared to after THA.

Male sex was a risk factor for revision due to infection after THA, as shown in some other studies (Ong et al. 2009, Pedersen et al. 2010), whereas yet other studies have not found this (Mahomed et al. 2003, Ridgeway et al. 2005). It also appears that males have a relatively high risk of revision due to infection—as compared to SSI. One reason may be different thresholds for referral or revision surgery, or the fact that surgery on males can cause a greater degree of surgical trauma and tissue damage (Franks and Clancy 1997, Borkhoff et al. 2008, Pedersen et al. 2010). There may also be differences in bacterial flora or carriage between men and women (Skramm et al. 2007).

The risk of infection increased with age, for both SSI and revision due to infection after THA, and this was also found to be the trend for the oldest HA patients. The exception was HAs in patients aged less than 60 years, who had greater than 3 times higher risk of revision due to infection than patients between 80 and 90 years of age. In Norway, the common policy is to use HA in young patients only if they have many risk factors for complications or have a short life expectancy. High age has been found to be an independent risk factor for SSI in some other studies (Ridgeway et al. 2005, Geubbels et al. 2006). In contrast, without adjustment for ASA class, high age was not found to be a risk factor for revision due to infection in a previous publication from the NAR involving THAs from the period 1987–2007 (Dale et al. 2009). A recent large Danish study, adjusted for co–morbidity, did not find age to be a risk factor (Pedersen et al. 2010). Having a primary THA at a young age may indicate co–morbidity, and thereby increased susceptibility to infection. Among very old patients the most healthy are selected to undergo THA, and may therefore have reduced susceptibility to infection compared to the average population at that age (Lie et al. 2000). Furthermore, a revision operation on hip arthroplasty is extensive surgery, and surgeons may sometimes choose a nonoperative approach in old and frail patients—an option that is not reported to the NAR and NHFR.

ASA class is a crude approximation of physical status, and works poorly at the individual level where there will be large inter–observer variability. In addition, different co–morbidities may have different effects on infection rates. However, ASA class has predictive value for complications in epidemiological studies like the present one, where the number of cases is large (Ridgeway et al. 2005, Bjørgul et al. 2010). Thus, all 3 registries have chosen the ASA classification as their measure of physical state. An ASA score higher than 1 had an increased risk of revision due to infection after THAs. This indicates that even minor co–morbidities may increase the risk of postoperative infection. For patients with an infected prosthesis, the treatment strategy may be nonoperative for higher ASA classes. In the latter case, some surgeons may choose lifelong antibiotic suppression rather than reoperation for a low–grade implant infection. This may be one explanation for why higher ASA scores had no increased risk of revision due to infection after HA in our study. It may also be that ASA class does not capture frailty in the elderly in a sufficient way in our study population (Makary et al. 2010).

We could not confirm findings from previous studies that longer duration of surgery is associated with higher risk of SSI and higher risk of revision due to infection after THA (Småbrekke et al. 2004, Ridgeway et al. 2005, Dale et al. 2009, Ong et al. 2009, Pedersen et al. 2010). However, duration of surgery less than 60 min was associated with higher risk of SSI after arthroplasty and risk of revision due to infection after HA. Similar findings were reported for SSI after revision arthroplasties, but not primary HA or THA, by Ridgeway (2005). Rapid surgery may result in inferior soft tissue treatment and hemostasis, thereby leading to increased risk of infection.

Primary arthroplasty performed as an emergency procedure after a femoral neck fracture increased the risk of both SSI and revision due to infection after THA. Ridgeway (2005) also found trauma to be a risk factor for SSI after THA. This may be due to local or systemic reactions to the trauma itself, to frailty of the patients, or to other confounders not reported to the registers. For HAs, there was no difference in the risk of revision due to infection between arthroplasty performed in the acute phase and planned surgery. A primary arthroplasty performed as planned surgery caused by a failed osteosynthesis is a reoperation, and may therefore resemble a revision arthroplasty more than a genuine primary arthroplasty. Revision arthroplasty and arthroplasty secondary to fractures are found to have higher susceptibility to infection (Berbari et al. 1998, Ridgeway et al. 2005, Jämsen et al. 2009a).

Cementless fixation had a higher risk of revision due to deep infection after THA, but not a higher risk of SSI. In Norway, nearly all cemented THAs are inserted with cement containing antibiotics (The Norwegian Arthroplasty Register 2010). Uncemented THAs can only be protected by antibiotic prophylaxis given systemically, and this was administered in nearly all hip arthroplasties in Norway over the study period (The Norwegian Arthroplasty Register 2010). Antibiotic eluted from cement is delivered locally, and protects the implant and periprosthetic tissue (Espehaug et al. 1997, Engesæter et al. 2003, Hendriks et al. 2005, Dale et al. 2009). This local antibiotic treatment appears to be less effective for protection against SSI.

The NNIS risk index is a combined surgery–related assessment tool developed to identify high–risk patients, and to evaluate the risk of SSI (Mangram et al. 1999). The NNIS index combines ASA class of greater than 2, duration of surgery longer than the 75th percentile for the procedure, and contamination of the wound. Considering our findings on ASA class and duration of surgery, and that arthroplasty is a clean procedure, the NNIS does not appear to be optimal for identification of patients who are at risk of infection after arthroplasty.

All data on completeness of case reporting to the NAR, the NHFR, and the NOIS, indicate that there would be minor selection bias in our study. The arthroplasties reported to the NOIS were similar, regarding the characteristics of patients and procedures, to the all–year–round registrations in the NAR and the NHFR. SSIs may have been underreported to the NOIS, just as revision due to infection has been to the NAR and other registers (Arthursson et al. 2005, Espehaug et al. 2006, Huotari et al. 2007, Jämsen et al. 2009b, Jämsen et al. 2010b). There is also a possibility of overestimation of SSI in surveillance systems, as superficial infections may be difficult to distinguish from aseptic wound complications (Walenkamp 2009). The lack of validation of endpoints is therefore a weakness in our study, even though we performed separate analyses on overall and deep infections without any major changes in risk assessment. This should be addressed in future studies. Surgical policy was also a possible confounder in the present study on the NHFR and the NAR, since different subgroups—such as patients with higher ASA classes and advanced age—may have been subject to different treatment strategies. For the NOIS and the NHFR, the number of cases included makes statistical power an issue when differences between subgroups are small or the numbers are low.

The 2 endpoints of infection in our study may reflect different types of infections, or at least different stages of infection. The NOIS is more likely to capture the acute virulent postoperative infections whereas the NAR/NHFR is more likely to capture either a more advanced stage of infection or more low–grade, late infections. This fact will affect the findings of incidence and risk patterns of infection, and it is important for the interpretation of results of studies with different definitions of infection and different follow–up.

The majority of SSIs were identified after discharge, which confirms earlier findings that post–discharge surveillance is important to capture the true incidence of SSI after hip replacement (Huenger et al. 2005, Huotari and Lyytikainen 2006, Mannien et al. 2008). Infection surveillance appears to reduce the incidence of SSI (Brandt et al. 2006), which is also the aim for the NOIS. The NAR has improved THA surgery in Norway over the last 25 years (Fevang et al. 2010). In 2005, the NHFR was established on the same basis with the same methodology. This has led to changes in the treatment of femoral neck fractures towards more use of HA (The Norwegian Hip Fracture Register 2010, Gjertsen et al. 2010). Adverse effects of such changes, such as infection, should be evaluated, which requires good–quality surveillance through registers like the NOIS, the NAR, and the NHFR.
